# Comparison of early onset sepsis and community-acquired late onset sepsis in infants less than 3 months of age

**DOI:** 10.1186/s12887-016-0618-6

**Published:** 2016-07-07

**Authors:** Shlomi Bulkowstein, Shalom Ben-Shimol, Noga Givon-Lavi, Rimma Melamed, Eilon Shany, David Greenberg

**Affiliations:** Pediatric Infectious Disease Unit, Soroka University Medical Center, P.O. Box 151, Beer-Sheva, 84101 Israel; Faculty of Health Sciences, Ben-Gurion University of the Negev, Beer Sheva, Israel; Neonatology Unit, Soroka University Medical Center, Beer Sheva, Israel

**Keywords:** Neonatal sepsis, Early vs. late onset, Infant, infection, Group B Streptococcus

## Abstract

**Background:**

We compared demographic and clinical characteristics of early-onset sepsis (EOS) and community-acquired late onset sepsis (CA-LOS) in infants.

**Methods:**

Our medical center is the sole hospital in southern-Israel, enabling incidence calculations. EOS (<7 days) and CA-LOS (7–90 days) episodes recorded between 2007 and 2013 were reviewed. Univariate and multivariate analyses were performed.

**Results:**

70 EOS and 114 CA-LOS episodes were recorded. The respective mean ± SD annual rates per 1,000 live-births were 0.66 ± 0.16 and 1.03 ± 0.23. Prematurity (42.9 % vs. 17.0 %), premature rupture of membranes (PROM; 22.9 % vs. 1.9 %), leukopenia (29.0 % vs. 11.6 %), thrombocytopenia (44.9 % vs. 14.3 %) and *Streptococcus agalactiae* infections (22.7 % vs. 8.1 %) were more common in EOS. Fever (25.4 % vs. 79.1 %) and *Streptococcus pneumoniae* infections (1.3 % vs. 12.9 %) were less common in EOS. In both groups, Gram-negative bacteria predominated (~60 %). Longer hospitalization duration (23.3 ± 25.1 vs. 10.3 ± 8.6 days) and higher case fatality rate (20.0 % vs. 5.3 %) were noted in EOS. Antibiotic resistance rates to empiric EOS and CA-LOS treatments were 0.0 % and 1.2 %, respectively.

In multivariate analysis, adjusting for prematurity and ethnicity, PROM, central line, low Apgar-score, low birth-weight, ventilation support and non-vaginal delivery were risk factors for EOS. Normal temperature, thrombocytopenia and leukopenia characterized EOS.

**Conclusion:**

EOS and CA-LOS rates were low in Jewish compared with Bedouin infants. EOS was characterized by higher rates of perinatal risk factors, *S. agalactiae* infections, normal temperature, thrombocytopenia, leukopenia and mortality, while fever and *S. pneumoniae* infections were common in CA-LOS. Current initial antibiotic regimens seem adequate, considering the susceptibility patterns of the isolated pathogens

**Electronic supplementary material:**

The online version of this article (doi:10.1186/s12887-016-0618-6) contains supplementary material, which is available to authorized users.

## Background

Sepsis is an important cause of morbidity and mortality among newborn infants [1, 2]. The overall incidence of neonatal sepsis ranges from one to 5 cases per 1,000 live births, and case fatality rates (CFRs) range from 2 % to 60 %. Both rates depend on multiple factors, such as pathogen distribution, gestational age, *Streptococcus agalactiae* (group B *Streptococcus*, GBS) carriage rates and prevalence of other common specific pathogens [1, 3, 4].

Sepsis in infants is often classified as being of early onset (EOS, 0–6 days) or late onset (LOS, 7–90 days) [5–8]. EOS is further divided into very early onset (V-EOS, 0–2 days) and other EOS (3–6 days), while LOS is sometimes further divided to age groups of 7–30 days and 31–90 days [5]. Another acceptable classification differentiates between hospital-acquired and community-acquired sepsis [5, 9–11].

Neonatal sepsis is initially treated by empiric antimicrobial therapy which is influenced by factors that include, among others, the likely etiologic agent and its known susceptibility patterns, community or hospital acquired infection and central nervous system (CNS) involvement [5]. EOS is more likely to be derived from vertical acquisition of microorganisms from the mother during labor [6], while LOS is more likely to be derived from horizontal transmission of pathogens from the infant's caregivers. Consequently, pathogens distribution and risk factors for EOS and LOS differ in many aspects [5, 6]. Moreover, risk factors for LOS are mainly defined for hospital-acquired cases [12]. However, data regarding risk factors and clinical manifestations of community-acquired LOS (CA-LOS) are scarce [9, 11, 13, 14]. Nevertheless, the empiric therapy used by most clinicians for the treatment of EOS and CA-LOS consists of penicillins in combination with aminoglycosides [6, 7].

In a previous study, conducted 20 years ago at the Soroka University Medical Center (SUMC), high gram-negative and very low GBS rates in both EOS and LOS were demonstrated [9]. The SUMC is the only hospital providing medical care in southern Israel, and therefore >95 % of deliveries (14,682 and 16,963 births/year in 2007 and 2013, respectively) and pediatric hospitalizations in the region take place at this institution, enabling incidence calculations.

We hypothesized that in the last twenty years, the epidemiology of neonatal sepsis in southern Israel has significantly changed.

The aim of this study was to compare risk factors, demographic and clinical characteristics of EOS and CA-LOS in southern Israel.

## Methods

### Study population and design

This was a retrospective, population-based study.

Data were derived from an ongoing prospective surveillance database, monitoring all positive blood or cerebrospinal fluid (CSF) samples from less than 90 day old infants. The database is managed by The Pediatric Infectious Disease Unit of the SUMC. Episodes of sepsis in infants identified between 2007 and 2013 were included.

The southern region of Israel (the Negev) has a heterogeneous population, consisting of ~80 % Jews (representing only ~50 % of all births), who live mainly in urban centers and a few rural communities, and 20 % Bedouin Arabs (~50 % of all births), who are in various stages of transition from semi-nomadism to settled modern day life. The two groups are different in many aspects, with the Bedouin population characterized as being of a lower socio-economic status, with higher rate of consanguineous marriage and fertility rate and a lesser use of prenatal care. The SUMC is the only medical center of the region and provides full primary and tertiary neonatal care to both populations [9, 15].

In southern Israel, >95 % of the children are born at the only medical center in the region. The proportion of children born at the SUMC in each ethnic group during the study period per year is approximately equal: 7507 Jewish children and 7174 Bedouin children, in 2013 [16].

### Case definitions

#### Sepsis

A positive blood or CSF culture with a recognized blood pathogen associated with appropriate clinical findings as judged by a senior neonatologist or a pediatric infectious disease specialist. Two successive positive blood cultures with the same antibiotic susceptibility pattern (in addition to appropriate clinical findings) were required for the diagnosis of coagulase negative *Staphylococcus* (CONS) infection.

#### EOS

An episode occurring in a newborn < 7 days old, resident of the region, with ≥1 positive blood or CSF cultures.

EOS was divided into very early onset (V-EOS, <3 days) and other EOS (3–6 days), we compared the risk factors, demographic, clinical and laboratory characteristics, distribution of pathogens and antibiotic susceptibility patterns of the two subgroups.

#### CA-LOS

An episode occurring in a non-hospitalized infant between the age of 7 and 90 days, resident of the region, with ≥1 positive blood or CSF cultures with a recognized blood pathogen. A new CA-LOS episodes had to be separated by >48 h from prior hospitalization discharge.

#### Meningitis

An episode with either positive bacterial CSF culture or positive blood culture with CSF pleocytosis (defined as >20 and >10 leukocytes/μl in infants <28 days and 28–90 days old, respectively).

#### Urosepsis

An episode with positive urine and blood culture for the same pathogens.

We defined risk factors for sepsis in accordance with those previously described [5]. The following risk factors were considered in our study: prematurity (<37 weeks); low birth weight (<2,000 g);premature rupture of membranes >16 h (PROM); low 5-min Apgar score (<8) and chorio-amnionitis (as defined by the treating physician).

### Data collection

Medical charts were reviewed retrospectively and the following clinical and laboratory data were extracted: age, gestational age, birth weight, sex, ethnicity (Jewish or Bedouin), maternal age, PROM, chorio-amnionitis, mode of delivery (vaginal, or instrumental delivery including cesarean section and vacuum), Apgar scores, temperature (highest and lowest recorded in the first day of each episode), blood count, cultures results, antibiotic susceptibilities, duration of hospitalization and mortality.

### Identification of isolates

Blood and CSF cultures were performed at the SUMC clinical microbiology laboratory. All blood cultures were processed using the Bactec 9240 system (Becton Dickinson, Franklin Lakes, NJ) and antibiotic susceptibility was determined using standard procedures [17].

For each episode, only one positive culture was accounted for. If identical bacteria were isolated simultaneously from blood and CSF the episode was defined as meningitis.

### Statistical analysis

Incidence was calculated as the number of episodes divided by the total population at risk during each study year. The age-specific and ethnicity-specific populations at risk were estimated according to the Israeli Central Bureau of Statistics reports [16]. Annual incidence rates were calculated as numbers of sepsis episodes per 1,000 live births.

Analysis of contingency data was conducted by the 2-tailed X^2^ test. Continuous variables were compared using the Student’s t test. *P* value of <0.05 was considered statistically significant.

Logistic regression models were used to evaluate potential risk factors, covariate and confounders. Variables implicated in the literature [5]: prematurity (<37 weeks); low birth weight (<2,000 g); premature rupture of membranes >16 h (PROM); low 5-min Apgar score (<8) and chorio-amnionitis and those that were statistically significant at the level of *P* < 0.1 in the univariate analyses were included in multivariate logistic regression models.

The study was approved by the Institutional Ethics Committees of the Soroka University Medical Center (SUMC).

## Results

**Fig. 1:**
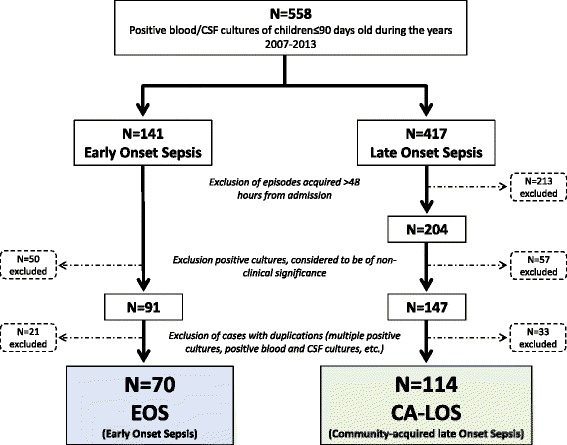
Flow chart of patient selection

During the study period, 558 positive blood and CSF cultures were identified. Of those, 141 (25.3 %) were from infants <7 days old (EOS) and 417 (74.7 %) from infants 7–90 days old (LOS). After exclusion of hospital-acquired episodes (LOS cultures obtained >48 h post-admission), duplications and cultures considered non-clinically significant, 70 EOS episodes and 114 CA-LOS episodes were included in the final analysis (Fig. 1). Of the 70 EOS episodes, 41 (58.6 %) occurred in the first 72 h (V-EOS), and 29 (41.4 %) more than 72 h after birth. Of the latter, 6 (20.7 %) occurred after hospital discharge from the newborn department. Of the 114 CA-LOS episodes, 45 (39.5 %), 39 (34.2 %) and 30 (26.3 %) occurred in the first, second and third months of life, respectively.

### Incidence of EOS and CA-LOS (Fig. 2)

**Fig. 2:**
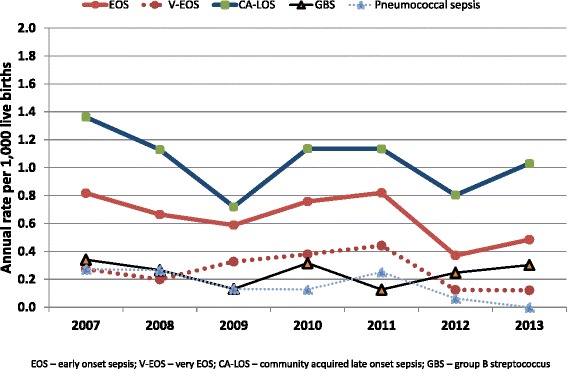
Incidence per 1,000 live births of V-EOS, EOS, CA-LOS, GBS and pneumococcal neonatal sepsis in southern Israel, 2007–2013

Mean (±SD) annual rates per 1,000 live births of EOS and CA-LOS were 0.66 ± 0.16 and 1.03 ± 0.23, respectively. The respective rates of V-EOS and pneumococcal sepsis were 0.26 ± 0.13 and 0.17 ± 0.13. The rates of EOS and CA-LOS caused by GBS were 0.17 ± 0.08 and 0.09 ± 0.04, respectively.

Non-significant declines in V-EOS, EOS, CA-LOS, GBS and pneumococcal sepsis rates were observed (comparing rates in 2013 vs. 2007), with respective declines of 38 % (*P* = 0.38), 44 % (*P* = 0.20), 38 % (*P* = 0.13), 31 % (*P* = 0.55) and 83 % (*P* = 0.10) during the study period.

### Demographic characteristics and risk factors for EOS vs. CA-LOS (Table 1)

**Table 1:** Demographic characteristics, risk factors and outcome of Early Onset Sepsis (EOS) (<7 days) vs. Community-acquired Late Onset Sepsis (CA-LOS) (7–90 days) in Southern Israel, 2007-2013

	EOS	CA-LOS	*P* value
	*N* = 70	*N* = 114	
Male (*n*, %)	46 (65.7 %)	63 (55.3 %)	0.161
Female (*n*, %)	24 (34.3 %)	51 (44.7 %)	
Jewish (*n*, %)	26 (37.1 %)	41 (36.0 %)	0.872
Bedouin (*n*, %)	44 (62.9 %)	73 (64.0 %)	
Non vaginal delivery (C/S or Vacuum)	33/70 (47.1 %)	28/112 (25.0 %)	0.002
Central line (*n*, %)	42/68 (61.8 %)	15/112 (13.4 %)	<0.001
Mechanical ventilation (*n*, %)	34/70 (48.6 %)	14/112 (12.5 %)	<0.001
Prematurity (<37 GA) (*n*, %)	30/70 (42.9 %)	19/112 (17.0 %)	<0.001
Birth weight < 2000 g. (*n*, %)	24/70 (34.3 %)	7/111 (6.3 %)	<0.001
PROM > 16 h (*n*, %)	16/70 (22.9 %)	2/108 (1.9 %)	<0.001
Chorio-Amnionitis	8/70 (11.4 %)	0/108 (0.0 %)	<0.001
Apgar score < 8 (*n*, %)	13/68 (19.1 %)	2/105 (1.9 %)	<0.001
Mean Hospitalization duration (days) ± SD	23.3 ± 25.1	10.3 ± 8.6	<0.001
Mortality (*n*, %)	14 (20.0 %)	6 (5.3 %)	0.002

*C/S*, cesarean section, *GA*, gestational age, *PROM*, premature rupture of membrane

Mean EOS and CA-LOS rates were higher in the Bedouin population than in the Jewish population; 0.77 ± 0.35 vs. 0.50 ± 0.29 for EOS and 1.29 ± 0.59 vs. 0.79 ± 0.32 for CA-LOS (*P* < 0.01, both). In contrast, mean GBS neonatal sepsis rate did not differ significantly in the Bedouin and the Jewish populations (0.19 ± 0.19 vs. 0.33 ± 0.08, *P* = 0.30).

Non-vaginal deliveries, central line exposure, mechanical ventilation, prematurity, low birth weight, PROM, chorio-amnionitis and low Apgar score were all significantly more common in EOS compared with CA-LOS.

### Clinical and laboratory characteristics (Table 2)

**Table 2:** Clinical Characteristics of Early Onset Sepsis (EOS) (<7 days) vs. Community-acquired Late Onset Sepsis (CA-LOS) (7–90 days) in Southern Israel, 2007-2013

	EOS	CA-LOS	*P* value
	*N* = 70	*N* = 114	
Meningitis (*n*, %)	10/69 (14.5 %)	23/112 (20.5 %)	0.306
Uro-sepsis (*n*, %)	7/69 (10.1 %)	35/112 (31.2 %)	0.001
Temperature ≥ 38 °C	16/63 (25.4 %)	87/110 (79.1 %)	<0.001
Temperature < 36 °C	3/63 (4.8 %)	13/110 (11.8 %)	0.123
WBC > 20,000 cells/μl	14/69 (20.3 %)	36/112 (32.1 %)	0.083
WBC < 5,000 cells/μl	20/69 (29.0 %)	13/112 (11.6 %)	0.005
PMN < 1,000 cells/μl (*n*, %)	10/69 (14.5 %)	6/111 (5.4 %)	0.037
PLT >450,000 cells/μl (*n*, %)	1/69 (1.4 %)	50/112 (44.6 %)	<0.001
PLT < 150,000 cells/μl (*n*, %)	31/69 (44.9 %)	16/112 (14.3 %)	<0.001

*WBC*, white blood cells, *PMN*, polymorphonuclear, *PLT*, platelets

While septicemia was more common in EOS, uro-sepsis was more common in CA-LOS and meningitis did not differ significantly between the two.

Temperature ≥38.0° (79.1 % vs. 25.4 %, *P* < 0.001) and hypothermia <36.0° (11.8 % vs. 4.8 %, *P* = 0.12) were more common in CA-LOS than in EOS.

Leukopenia <5,000/μl, neutropenia <1,000/μl and thrombocytopenia <150,000/μl were significantly more common in EOS, while leukocytosis > 20,000/μl was more common in CA-LOS (32.1 % vs. 20.3 %, *P* = 0.083).

### Distribution of pathogens (Table 3)

**Table 3:** Distribution of pathogens causing Early Onset Sepsis (EOS) (<7 days) vs. Community-acquired Late Onset Sepsis (CA-LOS) (7–90 days) in Southern Israel, 2007-2013

	EOS	CA-LOS	*P* value
	*N* = 75^a^	*N* = 124^a^	
Gram negative	44/75 (58.7 %)	78 (62.9 %)	0.552
*Escherichia coli*	26/75 (34.7 %)	36/124 (29.0 %)	0.406
*Klebsiella pneumoniae*	10/75 (13.3 %)	9/124 (7.3 %)	0.158
*Haemophilus influenzae*	1/75 (1.3 %)	8/124 (6.5 %)	0.157
*Pseudomonas spp.*	3/75 (4.0 %)	6/124 (4.8 %)	1.000
*Enterobacter cloacae*	0/75 (0.0 %)	5/124 (4.0 %)	0.159
Campylobacter spp.	0/75 (0.0 %)	3/124 (2.4 %)	0.292
*Neisseria meningitides*	0/75 (0.0 %)	3/124 (2.4 %)	0.292
Other	4/75 (5.3 %)	8/124 (6.5 %)	1.000
Gram positive	31/75 (41.3 %)	46/124 (37.1 %)	0.552
GBS	17/75(22.7 %)	10/124 (8.1 %)	0.004
*Streptococcus pneumoniae*	1/75 (1.3 %)	16/124 (12.9 %)	0.005
*Staphylococcus aureus*	4/75 (5.3 %)	12/124 (9.7 %)	0.275
*Enterococcus faecalis*	7/75 (9.3 %)	6/124 (4.8 %)	0.244
*Listeria monocytogenes*	2/75 (2.7 %)	0/124 (0.0 %)	0.141
*Streptococcus pyogenes*	0/75 (0.0 %)	2/124 (1.6 %)	0.528

^a^In 15 episodes (5 EOS and 10 CA-LOS episodes), mixed infection with 2 bacteria were identified

Overall, 199 bacteria were isolated in 184 episodes (in 15 episodes; 5 EOS and 10 CA-LOS episodes, mixed infection with 2 bacteria were identified).

Gram-negative bacteria comprised 58.7 % and 62.9 % of all EOS and CA-LOS episodes, respectively (*P* = 0.552), with *Escherichia coli* being the most common bacteria identified in both; 34.7 % and 29.0 % (*P* = 0.406), respectively.

In central line infections, *Escherichia coli* comprised 47.6 % of EOS compared with 13.3 % of CA-LOS (*p* = 0.03).

Among Gram-positive bacteria, GBS episodes were more common in EOS compared with CA-LOS (22.7 % vs. 8.1 %, *P* = 0.004). *Streptococcus pneumoniae* episodes were more common in CA-LOS (12.9 % vs.1.3 %, *P* = 0.005). The distribution of other bacteria was not significantly different in both groups.

### Antibiotic resistance (Additional file 1, Additional file 2)

In both groups, high proportions of Gram-negative bacteria were resistant to penicillins; 82.9 % in EOS and 67.6 % in CA-LOS to ampicillin and 35 % to piperacillin. None of the Gram positive EOS bacteria were resistant to penicillins, while 18.8 % of *S. pneumoniae* CA-LOS episodes were resistant to penicillin. Other Gram-positive CA-LOS episodes were all sensitive to penicillin.

In 100.0 % (104/104) of EOS and CA-LOS episodes, when tested, isolates were sensitive to amikacin, while 98.5 % (133/135) were sensitive to gentamicin. In 98.7 % (153/155) of all episodes, isolates were sensitive to3^rd^ generation cephalosporins.

Episodes with extended spectrum beta lactamase (ESBL) Gram negative bacteria were identified in 0.0 % and 1.3 % (*P* = 1.0) of EOS and CA-LOS, respectively.

While no isolated bacteria were found to be resistant to the empirical antibiotic treatment of piperacillin and amikacin for EOS, resistance rate to empirical CA-LOS antibiotic treatment of ampicillin and gentamicin was detected in 1.2 % (1/90) *E. coli* episodes). In 4 *Haemophilus influenzae* CA-LOS episodes, resistance to ampicillin was recorded, but susceptibility to gentamicin was not evaluated.

### Outcome

Mean hospitalization duration was longer (23.3 ± 25.1 vs.10.3 ± 8.6 days, *P* < 0.001) and case-fatality rate was higher in the EOS group (20 % vs.5.3 %, *P* = 0.002). Of the 14 EOS deaths, 11 episodes (78.8 %) were preterm infants (7 cases were extreme preterm infants, born <28 gestational age). In 6 episodes (42.9 %) PROM was recorded. The predominant pathogens were *E. coli* (8 episodes) and GBS (4 episodes).

In the 7 CA-LOS mortality cases, the predominant pathogens were *Staphylococcus aureus* and *S. pneumoniae* (2 episodes each).

### V-EOS (<3 days) vs. EOS during 3–6 days of age

Of the 70 EOS episodes, 41(58.6 %) were V-EOS episodes and 29 (41.4 %) occurred in neonates >72 h of age. No significant differences were found in most risk factors, demographic and clinical characteristics of the two groups, including prematurity, mean Apgar score and chorio-amnionitis rates; sex and ethnicity; temperature and leukocytes count; distribution of pathogens (excluding GBS) and antibiotic susceptibility pattern; and case fatality rates.

However, several differences between the two groups were observed: PROM (36.6 % vs. 3.4 %, *P* = 0.001) and GBS infections (36.4 % vs. 3.2 %, *P* = 0.001) were more common in V-EOS. In contrast, urosepsis (0.0 % vs.24.1 %, *P* = 0.001) and thrombocytopenia (22.5 % vs. 75.9 %, *P* < 0.001) were less common in V-EOS.

### Multivariate analysis

In a multivariate analysis adjusting for ethnicity and prematurity, the significant risk factors for EOS were PROM (OR:11.7), central-line (OR:10.9), 5 min Apgar score <8 (OR:9.9), low birth weight (OR:5.9), ventilation support (OR:5.6) and non-vaginal delivery (OR: 2.22). A trend for higher CFR in EOS was observed (OR: 2.8, *P* = 0.061) (Table 4).

**Table 4:** Multivariate analysis – adjusted for ethnicity and prematurity; odds ratio for early onset sepsis compared with community-acquired late onset sepsis

	Variable	OR	*P* value
Risk factors and outcome	PROM (>16 h)	11.68	0.002
	Central line	10.873	<0.0001
	Apgar < 8	9.858	0.004
	Low birth weight	5.882	<0.001
	Ventilation support	5.629	<0.0001
	Non vaginal delivery	2.216	0.019
	Mortality	2.8	0.061
	Gender	1.415	0.291
Clinical characteristics	36 °C < Fever < 38 °C	22.22	<0.0001
	PLT < 150,000	4.43	<0.0001
	Septicemia or meningitis (non-urosepsis)	3.98	0.003
	WBC <5,000	2.73	0.015
	WBC <20,000	2.22	0.04

*PROM*, premature rupture of membrane, *PLT*, platelets, *WBC*, white blood cells, *OR*, odds ratio

The following clinical characteristics were more common in EOS: normal temperature (absence of hypothermia or fever) (OR: 22.22), non-urosepsis clinical diagnosis (i.e. sepsis or meningitis, OR: 3.98), thrombocytopenia <150,000 cells/μl (OR: 4.43) and leukopenia <5000 cells/μl (OR: 2.73). In contrast, leukocytosis >20,000 cells/μl was less common in the EOS group (OR: 2.22).

## Discussion

In southern Israel, both EOS and CA-LOS rates were low in Jewish compared with Bedouin infants. EOS was characterized by higher rates of perinatal risk factors, *S. agalactiae* infections, normal temperature, thrombocytopenia, leukopenia and mortality. In contrast, fever and *S. pneumoniae* infections were more common in CA-LOS.

The finding of higher sepsis rates in the Bedouin population compared with the Jewish population is similar to a previous report from our medical center, 20 years ago [9]. In EOS, this was not related to the rate of GBS, but rather to the rate of other pathogens, mainly Gram-negative bacteria (*E. coli* and *Klebsiella* data not shown). This trend may be related to higher rates of prematurity, congenital malformations and lack of prenatal care in the Bedouin population [15] similarly to other developing populations [18–22].

Prematurity, PROM, low birth weight, chorio-amnionitis and low Apgar score were all more common in EOS vs. CA-LOS, similarly to previous reports [6, 23]. This is probably due to differences in pathogenesis, where in EOS episodes, bacteria are more likely to be acquired through the birth canal, while in CA-LOS the mode of infection is more likely to be by direct respiratory (droplets) and gastrointestinal contact of people surrounding the infant [6, 11, 13, 14].

Both temperature and complete blood cells counts (CBC) parameters differences between the two groups point to different capability of the host (the sick infant) to overcome infection. It is possible that neonates in the 1^st^ week of life are less capable of producing sufficient inflammatory response, reflected in the absence of temperature changes [24–26] and the absence of leukocytosis [27], possibly due to immature immune system [28–32]. This highlights the need for high degree of clinical suspicion for EOS even in the absence of temperature or CBC parameters changes. This is emphasized by the higher degree of disease severity (or incapability of the host to overcome the infection), evident by the significantly longer hospitalization duration and the higher mortality rates in the EOS group.

The mortality rates of neonatal sepsis ranged in different studies from 5 % to 60 %, depending upon the infecting agent, prematurity rates and co-morbidities [5, 23, 33].

The pathogen distribution observed in our study probably reflects differences in pathogenesis. In EOS infections, GBS and Gram-negative enteric bacteria predominated, while in CA-LOS, the rate of pneumococcal infections increased. Nevertheless, pathogens possibly acquired through the birth canal (e.g. GBS), are still present in CA-LOS. Several differences in pathogen distribution between our study and other reports are notable. First, GBS rates were relatively low in both the EOS and CA-LOS groups (mean rates per 1,000 of 0.17 and 0.09, respectively) in southern Israel compared with reports from Australia [34], The Netherlands [35] and other industrialized countries, such as the United States (US), United Kingdom (UK), Scotland, Northern Ireland, Germany, Finland and Portugal [36]. These low rates are comparable to previous reports from Israel [2, 37, 38]. Second, pneumococcal infections were common in the CA-LOS group in our study until recent years. However, pneumococcal rates declined in southern Israel since the year 2012, probably due to the introduction of pneumococcal conjugate vaccines and herd protection. Nevertheless, additional prospective studies should be conducted to confirm this observation. Third, *Listeria* infections were rare (only 2 EOS episodes throughout the study), although high maternal colonization rates in Israel have been previously reported [39].

These differences in pathogen distribution may be related to relatively low maternal GBS colonization rates [2], low virulence of specific bacterial clones (e.g. GBS, *Listeria*) and other unidentified environmental and epidemiological factors.

In contrast to other reports [5, 40], we did not find any CONS or fungi episodes in our study. This is presumably attributed to the exclusion of all hospital-acquired episodes in the current study and emphasizes the concept that these pathogens are related to nosocomial infections.

The current empiric treatment at the SUMC differs in the Neonatal Intensive Care Unit (NICU) from that administered in other settings (i.e. pediatrics wards). All isolates in both groups were sensitive to the combination of piperacillin and amikacin, which is the empiric treatment in our NICU. Furthermore, resistance rate to empirical antibiotic treatment outside the NICU setting in CA-LOS episodes (ampicillin and gentamicin) was relatively low. In addition, ESBL positive Gram-negative infections rates are low in our center. These findings confirm that the current empirical treatment is appropriate. However, it is important to notice that in CA-LOS, considerable penicillin resistance in *H. influenzae* and pneumococcal infections was noted.

The main limitation of our study lies in its retrospective nature. Consequently, some data regarding risk factors are missing (maternal GBS carriage, etc.) and we recognize the possibility of interpreting clinical significance bias.

The study main points of strengths include its unique ability to calculate the true incidence of infantile sepsis in our area, as well as its ability to compare this incidence between the Jewish and Bedouin populations. Additionally, the study relatively long duration (7 years) allows appreciation of specific pathogens rates fluctuations (e.g. GBS).

## Conclusions

Both EOS and CA-LOS rates were low in Jewish infants compared with those in Bedouin infants. EOS was characterized by higher rates of perinatal risk factors, *S. agalactiae* infections, normal temperature, thrombocytopenia, leukopenia and mortality. In contrast, fever and *S. pneumoniae* infections were more common in CA-LOS. Current initial antibiotic regimens seem adequate, considering the susceptibility patterns of the isolated pathogens.

## Abbreviations

CA-LOS, community-acquired late onset sepsis; CBC, blood cell count; CFR, case fatality rate; CNS, central nervous system; CONS, coagulase negative staphylococcus; CSF, cerebrospinal fluid; EOS, early onset sepsis; ESBL, extended spectrum beta lactamase; GBS, group B streptococcus; LOS, late onset sepsis; NICU, neonatal intensive care unit; PROM, premature rupture of membranes; SUMC, Soroka University Medical Center; V-EOS very early onset sepsis

## Additional files

Additional file 1: Antibiotic Resistance of Pathogens Causing Early Onset Sepsis (EOS) (<7 Days) vs. Late Onset Sepsis (7–90) In Southern Israel, 2007–2013. (DOCX 20 kb) Additional file 2: Antibiotic susceptibilities of pathogens causing EOS vs. CA-LOS in southern Israel, 2007–2013. (PPTX 78 kb)

## References

[1] Edwards MS, Baker CJ, Gershon AA, Hotez PJ, Katz SL (2004). Sepsis in the newborn. Krugman's Infectious Diseases of Children.

[2] Eidelman AI, Rudensky B, Turgeman D, Nubani N, Schimmel MS, Isacsohn M (1990). Epidemiology of group B streptococci colonization and disease in mothers and infants: update of ongoing 10-year Jerusalem study. Isr J Med Sci.

[3] Bailit JL, Gregory KD, Reddy UM, Gonzalez-Quintero VH, Hibbard JU, Ramirez MM (2010). Maternal and neonatal outcomes by labor onset type and gestational age. Am J Obstet Gynecol.

[4] Weston EJ, Pondo T, Lewis MM, Martell-Cleary P, Morin C, Jewell B (2011). The burden of invasive early-onset neonatal sepsis in the United States, 2005–2008. Pediatr Infect Dis J.

[5] Edwards MS, Baker CJ, Long SS, Pickering LK, Prober CG (2012). Bacterial infections in the neonate. Principles and Practice of Pediatric Infectious Disease.

[6] Polin RA, The Committee on Fetus and Newborn (2012). Management of neonates with suspected or proven early-onset bacterial sepsis. Pediatrics.

[7] Maayan-Metzger A, Barzilai A, Keller N, Kuint J (2009). Are the "good old" antibiotics still appropriate for early-onset neonatal sepsis? A 10 year survey. Isr Med Assoc J.

[8] Testoni D, Hayashi M, Cohen-Wolkowiez M, Benjamin DK, Lopes RD, Clark RH (2014). Late-onset bloodstream infections in hospitalized term infants. Pediatr Infect Dis J.

[9] Greenberg D, Shinwell ES, Yagupsky P, Greenberg S, Leibovitz E, Mazor M (1997). A prospective study of neonatal sepsis and meningitis in southern Israel. Pediatr Infect Dis J.

[10] Didier C, Streicher MP, Chognot D, Campagni R, Schnebelen A, Messer J (2012). Late-onset neonatal infections: incidences and pathogens in the era of antenatal antibiotics. Eur J Pediatr.

[11] Aggarwal R, Sarkar N, Deorari AK, Paul VK (2001). Sepsis in the newborn. Indian J Pediatr.

[12] Graham PL, Begg MD, Larson E, Della-Latta P, Allen A, Saiman L (2006). Risk factors for late onset gram-negative sepsis in low birth weight infants hospitalized in the neonatal intensive care unit. Pediatr Infect Dis J.

[13] Zaidi AK, Thaver D, Ali SA, Khan TA (2009). Pathogens associated with sepsis in newborns and young infants in developing countries. Pediatr Infect Dis J.

[14] Waters D, Jawad I, Ahmad A, Luksic I, Nair H, Zgaga L (2011). Aetiology of community-acquired neonatal sepsis in low and middle income countries. J Glob Health.

[15] Melamed Y, Bashiri A, Shoham-Vardi I, Furman B, Hackmon-Ram R, Mazor M (2000). Differences in preterm delivery rates and outcomes in Jews and Bedouins in Southern Israel. Eur J Obstet Gynecol Reprod Biol.

[16] Statistical Abstracts of Israel, 2013. No. 64. Central Bureau of Statistics. Jerusalem: State of Israel, 2013.

[17] CLSI. Performance standards for Antimicrobial Susceptibility Testing: Twenty-Fifth Informational Supplement. CLSI document M100-S19. Wayne, PA: Clinical and Laboratory Standards Institute; 2009.

[18] Moutquin JM (2003). Socio-economic and psychosocial factors in the management and prevention of preterm labour. BJOG.

[19] Hammond G, Langridge A, Leonard H, Hagan R, Jacoby P, DeKlerk N (2013). Changes in risk factors for preterm birth in Western Australia 1984–2006. BJOG.

[20] Ferguson SE, Smith GN, Salenieks ME, Windrim R, Walker MC (2002). Preterm premature rupture of membranes. Nutritional and socioeconomic factors. Obstet Gynecol.

[21] Sheridan E, Wright J, Small N, Corry PC, Oddie S, Whibley C (2013). Risk factors for congenital anomaly in a multiethnic birth cohort: an analysis of the Born in Bradford study. Lancet.

[22] Smith LK, Manktelow BN, Draper ES, Springett A, Field DJ (2010). Nature of socioeconomic inequalities in neonatal mortality: population based study. BMJ.

[23] Schuchat A, Zywicki SS, Dinsmoor MJ, Mercer B, Romaguera J, O'Sullivan MJ (2000). Risk factors and opportunities for prevention of early-onset neonatal sepsis: a multicenter case–control study. Pediatrics.

[24] Bekhof J, Reitsma JB, Kok JH, Van Straaten IH (2013). Clinical signs to identify late-onset sepsis in preterm infants. Eur J Pediatr.

[25] Voora S, Srinivasan G, Lilien LD, Yeh TF, Pildes RS (1982). Fever in full-term newborns in the first four days of life. Pediatrics.

[26] Hofer N, Zacharias E, Muller W, Resch B (2012). Performance of the definitions of the systemic inflammatory response syndrome and sepsis in neonates. J Perinat Med.

[27] Hornik CP, Benjamin DK, Becker KC, Benjamin DK, Li J, Clark RH (2012). Use of the complete blood cell count in early-onset neonatal sepsis. Pediatr Infect Dis J.

[28] Hornik CP, Benjamin DK, Becker KC, Benjamin DK, Li J, Clark RH (2012). Use of the complete blood cell count in late-onset neonatal sepsis. Pediatr Infect Dis J.

[29] Sadeghi K, Berger A, Langgartner M, Prusa AR, Hayde M, Herkner K (2007). Immaturity of infection control in preterm and term newborns is associated with impaired toll-like receptor signaling. J Infect Dis.

[30] Benitz WE, Han MY, Madan A, Ramachandra P (1998). Serial serum C-reactive protein levels in the diagnosis of neonatal infection. Pediatrics.

[31] Turner D, Hammerman C, Rudensky B, Schlesinger Y, Goia C, Schimmel MS (2006). Procalcitonin in preterm infants during the first few days of life: introducing an age related nomogram. Arch Dis Child Fetal Neonatal Ed.

[32] Benitz WE (2010). Adjunct laboratory tests in the diagnosis of early-onset neonatal sepsis. Clin Perinatol.

[33] Procianoy RS, Silveira RC, Mussi-Pinhata MM, Souza Rugolo LM, Leone CR, de Andrade Lopes JM (2010). Sepsis and neutropenia in very low birth weight infants delivered of mothers with preeclampsia. J Pediatr.

[34] Ireland S, Larkins S, Kandasamy Y (2014). Group B streptococcal infection in the first 90 days of life in North Queensland. Aust N Z J Obstet Gynaecol.

[35] Trijbels-Smeulders M, de Jonge GA, Pasker-de Jong PC, Gerards LJ, Adriaanse AH, van Lingen RA (2007). Epidemiology of neonatal group B streptococcal disease in the Netherlands before and after introduction of guidelines for prevention. Arch Dis Child Fetal Neonatal Ed.

[36] Le Doare K, Heath PT (2013). An overview of global GBS epidemiology. Vaccine.

[37] Ginsberg GM, Eidelman AI, Shinwell E, Anis E, Peyser R, Lotan Y (2013). Should Israel screen all mothers-to-be to prevent early-onset of neonatal group B streptococcal disease? A cost-utility analysis. Isr J Health Policy Res.

[38] German L, Solt I, Bornstein J, Ben-Harush S, Ben-Elishai M, Weintraub Z (2006). Is there an increase in the incidence of GBS carrier rates among pregnant women in northern Israel?. Harefuah.

[39] Elinav H, Hershko-Klement A, Valinsky L, Jaffe J, Wiseman A, Shimon H (2014). Pregnancy-associated listeriosis: clinical characteristics and geospatial analysis of a 10-year period in Israel. Clin Infect Dis.

[40] Cohen-Wolkowiez M, Moran C, Benjamin DK, Cotten CM, Clark RH, Benjamin DK (2009). Early and late onset sepsis in late preterm infants. Pediatr Infect Dis J.

