# Supplementary material for: Comparison of early onset sepsis and community-acquired late onset sepsis in infants less than 3 months of age
Source: BMC Pediatr. 2016 Jul 7;16:82. doi: 10.1186/s12887-016-0618-6 (PMC4936327; doi:10.1186/s12887-016-0618-6)
Supplement: Additional file 1: — Antibiotic Resistance of Pathogens Causing Early Onset Sepsis (EOS) (<7 Days) vs. Late Onset Sepsis (7–90) In Southern Israel, 2007–2013. (DOCX 20 kb) [file 12887_2016_618_MOESM1_ESM.docx]

**Additional file 1:** Antibiotic Resistance of Pathogens Causing Early Onset Sepsis (EOS) (<7 Days) vs. Late Onset Sepsis (7-90) In Southern Israel, 2007-2013

|  |  | **Early onset sepsis** | **Late onset sepsis** | ***P* value** |
| --- | --- | --- | --- | --- |
|  |  | **N= 75** | **N= 124** |  |
| **Penicillins** |  |  |  |  |
|  |  |  |  |  |
| **Ampicillin/penicillin** | ***Escherichia coli*** | 20/26 (76.9%) | 22/36 (61.1%) | 0.189 |
|  | **Overall gram Neg.** | 34/41 (82.9%) | 46/68 (67.6%) | 0.080 |
|  | ***Streptococcus pneumoniae*** | 0/1 (0.0%) | 3/16 (18.8%) | 1.000 |
|  | **Overall gram Pos.** | 0/27 (0.0%) | 3/34 (8.8%) | 0.248 |
|  | **All pathogens** | 34/68 (50.0%) | 49/102 (48.0%) | 0.802 |
|  |  |  |  |  |
| **Piperacillin** | ***Escherichia coli*** | 15/26 (57.7%) | 19/36 (52.8%) | 0.701 |
|  | **Overall gram neg** | 16/42 (38.1%) | 22/61(36.1%) | 0.834 |
|  |  |  |  |  |
| **Aminoglycosides** |  |  |  |  |
|  |  |  |  |  |
| **Amikacin** | ***Escherichia coli*** | 0/26 (0.0%) | 0/36 (0.0%) | -  - |
|  | **Overall gram Neg.** | 0/42 (0.0%) | 0/62 (0.0%) |  |
|  | **Overall gram Pos.** | - | - | - |
|  |  |  |  |  |
| **Gentamicin** | ***Escherichia coli*** | 1/26 (3.8%) | 1/36 (2.8%) | 1.000 |
|  | **Overall gram Neg.** | 1/42 (2.4%) | 1/63 (1.6%) | 1.000 |
|  | **Overall gram Pos.** | 0/12 (0.0%) | 0/18 (0.0%) | - |
|  | **All pathogens** | 1/54 (1.9%) | 1/81 (1.2%) | 1.000 |
|  |  |  |  |  |
| **Cephalosporins** |  |  |  |  |
| **(3^rd^generation)** |  |  |  |  |
|  |  |  |  |  |
| **Ceftriaxone/cefotaxime** | ***Escherichia coli*** | 0/26 (0.0%) | 1/36 (2.8%) | 1.000 |
|  | **Overall gram Neg.** | 0/41 (0.0%) | 2/68 (2.9%) | 0.526 |
|  | ***Streptococcus pneumoniae*** | 0/1 (0.0%) | 0/16 (0.0%) | - |
|  | **Overall gram Pos.** | 0/18 (0.0%) | 0/28 (0.0%) | - |
|  | **All pathogens** | 0/59 (0.0%) | 2/96 (2.1%) | 0.525 |
|  |  |  |  |  |
| **Ceftazidime** | ***Escherichia coli*** | 0/26 (0.0%) | 1/36 (2.8%) | 1.000 |
|  | **Overall gram Neg.** | 0/42 (0.0%) | 1/62 (1.6%) | 1.000 |
|  |  |  |  |  |
| **Vancomycin** |  |  |  |  |
|  | ***Streptococcus pneumoniae*** | 0/1 (0.0%) | 0/16 (0.0%) | - |
|  | **Overall gram Pos.** | 0/29 (0.0%) | 0/46 (0.0%) | **-** |
|  |  |  |  |  |
| **ESBL – gram neg. only** |  | 0/44 (0.0%) | 1/78 (1.3%) | 1.000 |
|  |  |  |  |  |

ESBL; extended-spectrum beta lactamase
