# Supplementary material for: Comparison of early onset sepsis and community-acquired late onset sepsis in infants less than 3 months of age
Source: BMC Pediatr. 2016 Jul 7;16:82. doi: 10.1186/s12887-016-0618-6 (PMC4936327; doi:10.1186/s12887-016-0618-6)
Supplement: Additional file 2: — Antibiotic susceptibilities of pathogens causing EOS vs. CA-LOS in southern Israel, 2007–2013. (PPTX 78 kb) [file 12887_2016_618_MOESM2_ESM.pptx]

## Slide 1
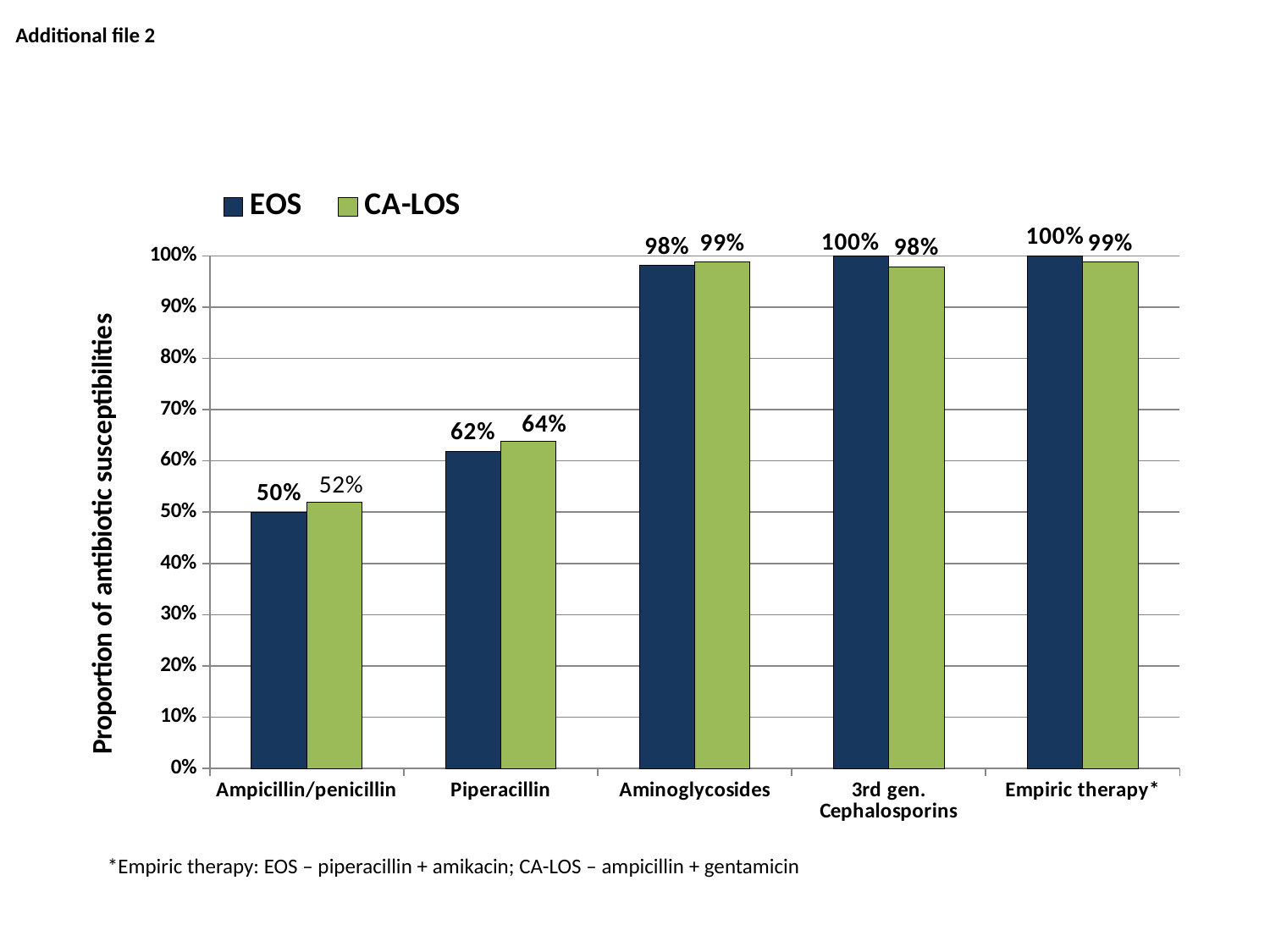

Additional file 2
### Chart
| Category | EOS | CA-LOS |
|---|---|---|
| Ampicillin/penicillin | 0.5 | 0.52 |
| Piperacillin | 0.619 | 0.639 |
| Aminoglycosides | 0.981 | 0.988 |
| 3rd gen. Cephalosporins | 1.0 | 0.979 |
| Empiric therapy* | 1.0 | 0.988 |*Empiric therapy: EOS – piperacillin + amikacin; CA-LOS – ampicillin + gentamicin
